# GPs’ perceptions of unintelligible text or codes in discharge summaries as potential threats to patient safety: a nationwide survey

**DOI:** 10.3399/BJGPO.2025.0079

**Published:** 2026-05-06

**Authors:** Thorbjørn Hougaard Mikkelsen, Jesper Bo Nielsen, Maria Munch Storsveen, Jens Søndergaard

**Affiliations:** 1 Research Unit of General Practice, Department of Public Health, University of Southern Denmark, Odense, Denmark

**Keywords:** patient safety, general practice, patient discharge, surveys and questionnaires, primary health care, Denmark

## Abstract

**Background:**

Follow-up by the GP after the patient’s discharge from hospital is important for patient safety and reduces the risk of readmission to hospital. Hence, it is important that discharge summaries provide effective discharge communication to general practice.

**Aim:**

To investigate whether GPs perceive incomprehensible text elements in discharge summaries as a potential threat to patient safety.

**Design & setting:**

Nationwide survey among a representative sample of Danish GPs.

**Method:**

A questionnaire was prepared for GPs based on background material, focus group interviews, and discussions with relevant professionals. It was subsequently pilot tested by fellow researchers and GPs and revised before the presently reported survey.

**Results:**

Of the 310 participating GPs, 201 (64.8%) ‘totally agree’ or ‘partially agree’ that discharge summaries contain incomprehensible text or codes, causing them concern about missing important information in discharge summaries. In total, 238 (76.8%) participants ‘totally agree’ or ‘partially agree’ that incomprehensible text or codes in discharge summaries are a threat to patient safety.

**Conclusion:**

The survey reveals that most GPs find discharge summaries contain incomprehensible text or codes, which raises concerns about missing critical information that may pose a threat to patient safety.

## How this fits in

Follow-up by the GP after the patient’s discharge from hospital is important for patient safety and reduces the risk of readmission to hospital. Studies show that failures occur in the processing of requested actions in almost half of all discharge summaries. This study adds that most GPs experience discharge summaries that contain incomprehensible text or codes, causing them concern about missing important information. In addition, most GPs think that incomprehensible text or codes in discharge summaries are a threat to patient safety.

## Introduction

Studies have repeatedly examined the communication between healthcare sectors.^1–6^ Transitions within the healthcare system, such as hospital discharges, can lead to information gaps, misunderstandings among healthcare professionals, ambiguity regarding treatment responsibilities, medication errors, and interruptions in care continuity.^7–11^ Consequently, prioritising effective discharge communication between healthcare professionals is crucial, particularly in reducing the risk of adverse events.^3,12^

This national survey among GPs investigates whether incomprehensible text in discharge summaries is considered a potential threat to patients’ safety. In Denmark, the vast majority of patients are registered with a GP, who acts as gatekeeper and ensures coordination of care.^13^ Danish hospitals and GPs are organised in geographical regions. Each of the five Danish regions has its own electronic medical record (EMR) system in which discharge letters are made; however, Region Zealand and the Capital Region of Denmark have the same EMR system. The GPs use eight different types of EMR system, each of which displays discharge summaries in different ways. These aspects, in combination with personal preferences, such as the type of clinic, may influence the GPs’ perceptions of the discharge summaries.

Following discharge from the hospital, responsibility for the patient’s care is transferred from the hospital to the GP.^14^ Follow-up by the GP after the patient’s discharge from hospital is important for patient safety and reduces the risk of hospital readmission.^3,15,16^ However, GPs often have little time to read discharge summaries,^17–19^ and failures have been reported in the processing of requested actions stated in them.^19–21^ One study found such failures in the processing of almost half of the requested actions.^20^ In Denmark, unintelligible text or codes in discharge letters that are automatically generated by a hospital’s EMR system can be a challenge for GPs to understand and navigate.^22^

This study aimed to investigate whether GPs worry about missing important information owing to incomprehensible text elements in discharge summaries and if they perceive them as a potential threat to patient safety.

## Method

### Study population

A questionnaire was sent to a representative sample of GPs in Denmark by the Danish Organisation of General Practitioners (PLO), the professional body for all GPs in Denmark. The questionnaire survey was carried out from 5 January to 26 January 2021. Non-responders received one reminder after 2 weeks. It took approximately 15 minutes to complete the questionnaire. The GPs were remunerated in accordance with instructions laid down by the Danish College of General Practitioners (DSAM), corresponding to DKK 206.43 (approximately 23.50 GBP) for answering the questionnaire.

The questionnaire was distributed and collected by PLO, who dispatched it to 800 randomly chosen GPs listed in the PLO’s membership database, which contains ≥99% of all Danish GPs. Two GPs were immediately deleted owing to inactive mailboxes, leaving 798, as presented in Table 1. Of these, two GPs were removed: one was not working in general practice at that time, and another was removed owing to participation in this project, leaving 796 eligible GPs. This approach ensured an equitable distribution across the entire group of GPs in terms of sex, age, region, and practice type. At the time of the survey, PLO’s membership databases comprised 3473 GPs, showing that approximately one in four was invited. As can be seen from Table 1, the sample and the responders closely resemble the composition of Danish GPs regarding sex, age, and geographic region.

**Table 1.** Characteristics of responding GPs (*N* = 310) compared with all Danish GPs

**Characteristic**	**Population of Danish GPs (N = 3473)**		**Sample of GPs (*N =* 798)**			**Responding GPs (*N =* 310)**		
	***n***	**%**	***n***	**%**	**95% CI**	***n***	**%**	**95% CI**
**Sex**								
Male	1499	43.2	340	42.6	39.2 to 46.1	144	46.5	40.8 to 52.2
Female	1974	56.8	458	57.4	53.9 to 60.9	166	53.5	47.8 to 59.2
**Geographical region**								
The Capital Region of Denmark	1064	30.6	248	31.1	27.9 to 34.4	88	28.4	23.4 to 33.8
The Central Denmark Region	441	12.7	87	10.9	8.8 to 13.3	41	13.2	9.7 to 17.5
The North Denmark Region	819	23.6	200	25.1	22.1 to 28.2	79	25.5	20.7 to 30.7
Region Zealand	851	24.5	197	24.7	21.7 to 27.8	78	25.2	20.4 to 30.4
Region of Southern Denmark	298	8.6	66	8.3	6.5 to 10.4	24	7.7	5.0 to 11.3
**Age range, years**								
30–40	320	9.2	81	10.2	8.1 to 12.5	30	9.7	6.6 to 13.5
41–50	1421	40.9	316	39.6	36.2 to 43.1	119	38.4	33.0 to 44.1
51–60	989	28.5	229	28.7	25.6 to 32.0	95	30.6	25.6 to 36.1
61–70	660	19.0	154	19.3	16.6 to 22.2	61	19.7	15.4 to 24.6
>70	83	2.4	18	2.3	1.3 to 3.5	5	1.6	0.5 to 3.7

### Questionnaire

The items in the questionnaire were based on aspects identified in material such as legal obligations on how to prepare a discharge summary, as well as in focus group interviews (FGI) with GPs and hospital physicians.

The knowledge from the background material was used to produce a semi-structured interview guide based on open-ended prompt questions aiming to explore the physicians’ experiences with the discharge summaries.^23^ The FGIs were transcribed and analysed in NVivo. We primarily used a phenomenological inductive approach focusing on the informants’ experiences, perceptions, understandings, and ideas.^23^ We also applied a deductive analytic strategy based on the themes of the interview guide.^23^ This knowledge, in combination, was the basis for the final items in the questionnaire.

In this way we prepared the questionnaire for the GPs based on background material, FGIs, and discussions with the project advisory board, including representatives from the five Danish regions responsible for hospitals and health care; PLO; MedCom, which provides digital solutions providing healthcare information to healthcare providers; and the Danish Patient Safety Authority. The questionnaire was constructed with answers on a five-point Likert scale, pilot tested by fellow researchers and GPs, and revised before the survey. See Supplementary Information S1 for the final survey.

### Statistical analysis

Unadjusted and adjusted analyses were used. We adjusted for the influence of region, EMR system, sex, age group (≤55 years versus >55 years), type of clinic (solo versus multi), and whether the clinic employed nurses. The rationale for including the list of covariates in the adjusted multivariate models is that sex and age could possibly affect the GP’s willingness to embrace new electronic solutions. The size of the clinic and whether it employs nurses may influence how accustomed the GPs are to taking advice from colleagues and hence how they perceive recommendations from other healthcare professionals. We applied an adjusted logistic regression, adjusting for geographical region, the EMR system provider used by the clinics, sex, age group, type of clinic, and whether the clinic employed ≥1 nurses. A *P*-value of ≤0.05 was considered statistically significant. Stata (version 18) was used for analysis.

## Results

Among the 796 GPs, 310 (38.9%) responded. There were no statistically significant differences between the population of Danish GPs, the sample, and the responding GPs in relation to sex, geographical region, or age group (Table 1).

The distribution of EMR system was approximately the same among Danish GP clinics and the replying GPs (Table 2).

**Table 2.** Distribution of EMR system in general practice clinics and among the GPs who answered the questionnaire

**EMR system used by clinics**	**Distribution of system among Danish GP clinics (*N =* 1716)**		**Distribution of system among replying GPs (*N =* 310)**	
	***n***	**%**	***n***	**%**
1	725	42.2	133	42.9
2	436	25.4	87	28.1
3	201	11.7	27	8.7
4	239	13.9	46	14.8
5–7^a^	114	6.6	17	5.5
8	1	0.06	0	0.0

^a^EMR systems 5–7 are merged owing to few users. EMR = electronic medical record.

Of the 310 participating GPs, 201 (64.8%) ‘totally agree’ or ‘partially agree’ that discharge summaries contain incomprehensible text or codes, causing them concern about missing important information, whereas 49 (15.8%) ‘neither agree nor disagree’, and 48 (15.5%) and 12 (3.9%) ‘partially disagree’ and ‘completely disagree’, respectively. When asked, 238 (76.8%) ‘totally agree’ or ‘partially agree’ that incomprehensible text or codes in discharge summaries are a threat to patient safety; 39 (12.6%) ‘neither agree nor disagree’, 26 (8.4%) ‘partially disagree’, and seven (2.3%) ‘completely disagree’ (Table 3).

**Table 3.** Responses to statements (*N* = 310)

**Statement**	**‘Totally agree’**	**‘Partially agree’**	**‘Neither agree nor disagree’**	**‘Partially disagree’**	**‘Completely disagree’**
	***n*** **(%)**	***n*** **(%)**	***n*** **(%)**	***n*** **(%)**	***n*** **(%)**
Discharge summaries contain incomprehensible text or codes that make me worry about missing important information	84 (27.1)	117 (37.7)	49 (15.8)	48 (15.5)	12 (3.9)
Incomprehensible text or codes in discharge summaries are a threat to patient safety	127 (41.0)	111 (35.8)	39 (12.6)	26 (8.4)	7 (2.3)

While 201 (64.8%) GPs reported worrying about missing important information because of incomprehensible text or codes, more GPs (*n* = 238, 77%) stated that incomprehensible text or codes were a threat to patient safety. These numbers show that GPs believe it is a significant issue regarding patient safety.

When region, EMR system used by the clinics, sex, age groups, type of clinic, and whether the clinic employed nurses were evaluated in an adjusted model, the only statistically significant difference in responses was seen between GPs in Region Zealand compared with the reference group (Central Denmark Region) in the perception of whether discharge summaries containing incomprehensible text or codes cause them concern about missing important information (Table 4).

**Table 4.** Responses to the statement: ‘Discharge summaries contain incomprehensible text or codes that make me worry about missing important information’

**Category**	***N***	**‘Totally agree' or 'partially agree',** ***n*** **(%)**	**‘Neither agree nor disagree', 'partially disagree', or 'completely disagree',** ***n*** **(%)**	**OR (95% CI)** ^**a**^	**Adjusted OR (95% CI)** ^**b**^
**Geographical region**					
The Central Denmark Region	78	46 (59.0)	32 (41.0)	1	1
The Capital Region of Denmark	88	64 (72.7)	24 (27.3)	1.86 (0.97 to 3.56)	1.92 (0.94 to 3.94)
The North Denmark Region	24	16 (66.7)	8 (33.3)	1.39 (0.53 to 3.64)	1.35 (0.50 to 3.61)
Region Zealand	41	35 (85.4)	6 (14.6)	4.06 (1.53 to 10.79)^c^	3.93 (1.46 to 10.62)^d^
Region of Southern Denmark	79	40 (50.6)	39 (49.4)	0.71 (0.38 to 1.34)	0.74 (0.40 to 1.40)
**EMR system used by clinics**					
1	133	78 (58.6)	55 (41.4)	1	1
2	87	61 (70.1)	26 (29.9)	1.65 (0.93 to 2.94)	1.43 (0.78 to 2.60)
3	27	17 (63.0)	10 (37.0)	1.20 (0.51 to 2.82)	0.84 (0.33 to 2.13)
4	46	32 (69.6)	14 (30.4)	1.61 (0.79 to 3.30)	1.12 (0.52 to 2.41)
5–7^e^	17	13 (76.5)	4 (23.5)	2.29 (0.71 to 7.42)	1.48 (0.38 to 5.77)
**Sex**					
Male	144	100 (69.4)	44 (30.6)	1	1
Female	166	101 (60.8)	65 (39.2)	0.68 (0.43 to 1.10)	0.67 (0.41 to 1.11)
**Age, years**					
≤55	194	123 (63.4)	71 (36.6)	1	1
>55	116	78 (67.2)	38 (32.8)	1.18 (0.73 to 1.93)	1.08 (0.64 to 1.84)
**Type of clinic**					
Multi^f^	235	150 (63.8)	85 (36.2)	1	1
Solo^g^	75	51 (68.0)	24 (32.0)	1.20 (0.69 to 2.10)	1.02 (0.54 to 1.92)
**≥1 nurses employed in the practice**					
Yes	275	178 (64.7)	97 (35.3)	1	1
No	35	23 (65.7)	12 (34.3)	1.04 (0.50 to 2.19)	0.89 (0.39 to 1.98)

^a^Crude prevalence ratio is the unadjusted prevalence ratio. ^b^Adjusted logistic regression. Adjusted for geographical region, EMR system used by the clinics, sex, age group, practice type, and ≥1 nurses employed in the practice. ^c^ *P *= 0.005. ^d^ *P *= 0.007. ^e^EMR systems 5–7 are merged owing to few users. ^f^Partnership clinics. ^g^Solo, shared single, and groups of solo GPs sharing clinic facilities. EMR = electronic medical record. OR = odds ratio.

When region, EMR system used by the clinics, sex, age groups, type of clinic, and whether the clinic employed nurses were evaluated in an adjusted model, the only significant difference in responses was seen between GPs in Region Zealand compared with the reference group (Central Denmark Region) in the perception of whether incomprehensible text or codes in discharge summaries were perceived as a threat to patient safety (Table 5).

**Table 5.** Responses to the statement: ‘Incomprehensible text or codes in discharge summaries are a threat to patient safety’

**Category**	***N***	**‘Totally agree' or 'partially agree',** ***n*** **(%)**	**‘Neither agree nor disagree', 'partially disagree', or 'completely disagree',** ***n*** **(%)**	**OR (95% CI)** ^**a**^	**Adjusted OR (95% CI)** ^**b**^
**Geographical region**					
The Central Denmark Region	78	60 (76.9)	18 (23.1)	1	1
The Capital Region of Denmark	88	70 (79.5)	18 (20.5)	1.17 (0.56 to 2.45)	1.25 (0.57 to 2.75)
The North Denmark Region	24	19 (79.2)	5 (20.8)	1.14 (0.37 to 3.49)	0.97 (0.31 to 3.08)
Region Zealand	41	39 (95.1)	2 (4.9)	5.85 (1.28 to 26.69)^c^	6.63 (1.52 to 28.92)^d^
Region of Southern Denmark	79	50 (63.3)	29 (36.7)	0.52 (0.26 to 1.04)	0.53 (0.26 to 1.07)
**EMR system used by the clinics**					
1	133	97 (72.9)	36 (27.1)	1	1
2	87	71 (81.6)	16 (18.4)	1.65 (0.85 to 3.20)	1.35 (0.67 to 2.73)
3	27	19 (70.4)	8 (29.6)	0.88 (0.35 to 2.19)	0.75 (0.29 to 1.93)
4	46	38 (82.6)	8 (17.4)	1.76 (0.75 to 4.14)	1.36 (0.56 to 3.32)
5–7^e^	17	13 (76.5)	4 (23.5)	1.21 (0.37 to 3.95)	0.94 (0.24 to 3.74)
**Sex**					
Male	144	114 (79.2)	30 (20.8)	1	1
Female	166	124 (74.7)	42 (25.3)	0.78 (0.46 to 1.33)	0.72 (0.41 to 1.26)
**Age, years**					
≤55	194	152 (78.4)	42 (21.6)	1	1
>55	116	86 (74.1)	30 (25.9)	0.79 (0.46 to 1.36)	0.77 (0.43 to 1.39)
**Type of clinic**					
Multi^f^	235	182 (77.4)	53 (22.6)	1	1
Solo^g^	75	56 (74.7)	19 (25.3)	0.86 (0.47 to 1.57)	0.93 (0.46 to 1.87)
**≥1 nurses employed in the clinic?**					
Yes	275	215 (78.2)	60 (21.8)	1	1
No	35	23 (65.7)	12 (34.3)	0.53 (0.25 to 1.14)	0.47 (0.21 to 1.07)

^a^Crude prevalence ratio is the unadjusted prevalence ratio. ^b^Adjusted logistic regression. Adjusted for geographical region, EMR system used by the clinics, sex, age group, practice type, and ≥1 nurses employed in the practice. ^c^ *P *= 0.005. ^d^ *P *= 0.007. ^e^EMR systems 5–7 are merged owing to few users. ^f^Partnership clinics. ^g^Solo, shared single, and groups of solo GPs sharing clinic facilities. EMR = electronic medical record. OR = odds ratio.

## Discussion

### Summary

When discharge summaries were introduced in general practice in Denmark, the chair of the PLO stated that unintelligible text or codes stemming from automatically generated codes in discharge letters can be a challenge for GPs.^22^ The extent of the problem and whether unintelligible text or codes in reality pose a challenge to patient safety remains unknown. However, this study reveals that most GPs believe that incomprehensible text or codes in discharge summaries are a threat to patient safety. Additionally, most GPs find that discharge summaries contain such text or codes, raising concerns about missing critical information. These issues are prevalent across all regions of Denmark. However, GPs in Region Zealand more often than those in other regions report encountering incomprehensible text or codes, which heightens their concern about missing important information. Consequently, most GPs perceive these elements in discharge summaries as a threat to patient safety.

### Strengths and limitations

The purpose of the discharge summary is to ensure better knowledge transfer to general practice and to enhance patient safety. Our questionnaire does not provide definitive clarity over whether this intended purpose is achieved, as it only captures the experiences of GPs. However, a strength of this study is that it offers a valid perspective from the primary recipients of discharge letters (GPs), though it would be desirable to explore perspectives from both primary and secondary care.^3,24,25^ While 201 (64.8%) GPs worried about missing important information because of incomprehensible text or codes, even more GPs (*n* = 238, 77%) stated that incomprehensible text or codes were a threat to patient safety. These findings show that GPs believe it is a significant issue regarding patient safety, as well as for their professional handling of discharge summaries. Additionally, it is noteworthy that the sample and responders closely mirror the composition of Danish GPs. Although the response rate of 38.9% is a limitation, it is not uncommon for surveys in general practice.^26–28^ This may result in biased outcomes, as GPs with strong opinions about the new discharge letters are likely the ones most inclined to respond, regardless of whether their views are positive or negative. With the data being sampled in 2021, it is relevant to question if data are still valid in 2025. However, a strength of this study is that guidelines for filling out the text box; the EMR system used to handle the discharge summaries; and the user interface have not changed since the data were collected, except that the EMR system used in Region Zealand and the Capital Region of Denmark have shortened the discharge summaries and removed unnecessary information that may have removed some of the incomprehensible text.

This indicates that while automatically generated codes may ease the production of discharge summaries, it may on the other hand create incomprehensible text or codes, causing the GP to worry about missing important information in discharge summaries, which is believed to be a threat to patient safety. These findings further indicate that implementing new IT systems requires thorough pre-launch testing, including assessment of front-end feasibility.

### Comparison with existing literature

In 2019, Denmark implemented a new type of discharge summary that included a recommendation text box.^29^ The results have been positive, showing that these discharge summaries ensure that GPs know which discharge letters to act on immediately after a patient has been discharged, thus reducing information overload.^29^ In addition, the recommendation text box in the discharge summary is easy to find, and provides brief and precise information about the recommended follow-up.^30^ This study adds that despite much positivity, most GPs find that discharge summaries contain incomprehensible text or codes, causing them concern about missing important information, and they consider this to be a threat to patient safety.

The quality of precise information transfer between healthcare professionals has been established elsewhere as an important aspect of the discharge of older patients to primary health care.^31^ Other studies suggest that adverse events occur in 19%–28% of hospitalised patients in the initial 30 days after discharge^32,33^ and are typically attributed to medication (72%), medical errors (16%), and hospital-acquired conditions (11%).^32,34^ Another study found that incomplete elements of discharge summaries increased readmission risk for patients with heart failure.^35^ This indicates that adverse events stemming from obscured, incomplete, or missing information in discharge summaries needs continued focus.

In addition, less tangible ‘soft effects’ of suboptimal communication have been reported elsewhere, including aspects such as inefficient use of GP time, reduced quality of care, and compromised continuity of care, as well as increased decisional uncertainty.^25^

In combination, our results indicate that further research is needed. Region Zealand and the Capital Region of Denmark use the same EMR system.^36^ Hence, it could be expected that the results would be similar. However, in our study, 64 (72.7%) GPs in the Capital Region of Denmark totally or partially agreed that discharge summaries contain incomprehensible text or codes, which caused them concern about missing important information in the discharge summaries, compared with the 35 (85.4%) GPs who had the same view in Region Zealand.

Similarly, 70 (79.5%) GPs in the Capital Region of Denmark totally or partially agreed that incomprehensible text or codes are a threat to patient safety, compared with 39 (95.1%) GPs in Region Zealand. Since the EMR system is the same, these numbers indicate that results within the two regions are influenced by other aspects.

The differences might be caused by a different introduction to and use of EMR system between the two regions. Automatically generated codes, which may only be used if they are relevant for the GP,^22,37^ might account for some of the difference if not used properly, as reported elsewhere;^29^ for example, by the chairman of the PLO back in 2019.^22^

The differences may also be influenced by a different implementation process: stepwise was introduced into the hospitals in the Capital Region of Denmark between May 2016 and May 2017, and into all hospitals in Region Zealand in November 2017.

The time since the introduction may also be a factor influencing our results in the two geographical regions. The Capital Region of Denmark has, among other things, been criticised for their implementation process and introducing an EMR system that limits job satisfaction, leading to unsafe patient treatment and reducing humanity in the meeting between patient and healthcare professionals. Region Zealand seems to have been less exposed to this specific criticism.^38^ This may have caused the Capital Region of Denmark to improve their implementation process to a greater extent than Region Zealand, perhaps leading to a better performance in our survey. In addition, Region Zealand employs more physicians educated in another country than the Capital Region of Denmark,^39^ hence a lack of language proficiency may also have influenced the results.

### Implications for research and practice

The survey findings reveal that most GPs find that discharge summaries contain incomprehensible text or codes, raising concerns about missing crucial information. Additionally, most GPs assess that such text or codes in discharge summaries pose a threat to patient safety. Our findings highlight the importance of involving GPs, as the recipients of discharge summaries, in evaluations of their quality and relevance alongside hospital physicians who write them, as proposed elsewhere.^25^ This collaboration could facilitate a more seamless transition between healthcare sectors.

The survey findings also indicate significant regional differences. Future studies should investigate incomprehensible text or codes in greater detail.

## Data

The datasets used in this study are not publicly available due to regulations from the Danish Data Protection Agency.
